# The Impact of Rural Clinical Placements on Medical Students’ Career Choices: A Systematic Review

**DOI:** 10.34172/ijhpm.9328

**Published:** 2026-05-05

**Authors:** Marc-Antoine Schirm, Jan Chrusciel, Maxime Thorigny, Aline Hurtaud, Nathalie Bednarek, Stephane Sanchez

**Affiliations:** ^1^Public Health Department, Champagne Sud Hospital, Troyes Hospital, Troyes, France.; ^2^Department of Neonatology, Reims University Hospital Alix de Champagne, Reims, France.; ^3^Department of General Practice, Faculty of Medicine, University of Reims Champagne-Ardenne, Reims, France.; ^4^Laboratory REGARDS, Research Unit 6192, University of Reims Champagne-Ardenne, Reims, France.; ^5^Research Unit 3797 VieFra, Faculty of Medicine, University of Reims Champagne-Ardenne, Reims, France.

**Keywords:** Rural Healthcare, Medical Practice, Underserved Areas, Medical Training, Meta-analysis

## Abstract

**Background:**

The uneven distribution of physicians between urban and rural areas remains a significant public health challenge. Rural clinical placements during medical training are increasingly viewed as a potential lever for enhancing the appeal of rural areas. The aim of this systematic review, supported by a meta-analysis, was to evaluate the real impact of rural placements on medical graduates’ decisions to practice in rural locations.

**Methods:**

A systematic search was conducted across four databases (PubMed, Embase, Web of Science, and Cochrane Library) covering the data available up to June 2024. Included studies evaluated the impact of rural clinical placements on the professional practice location of medical students or early-career physicians. A random-effects meta-analysis was performed to estimate the relative risk (RR) of rural practice. Additional variables analyzed included practice duration, placement length, participants’ rural background, their satisfaction with the placement, and their stated intention to practice in a rural area.

**Results:**

A total of 62 studies were selected, with 28 contributing to the meta-analysis, encompassing over 330 000 participants. The pooled analysis showed that students who completed a rural placement were more than twice as likely to establish practice in a rural area compared to their non-exposed peers (RR=2.683; 95% CI [confidence interval]: 2.255–3.192). This effect was further amplified by factors such as rural background, extended placement duration, and a satisfactory placement experience. A key strength of this review is that it synthesizes actual workforce outcomes rather than relying solely on stated intentions.

**Conclusion:**

Rural clinical placements have a significantly positive impact on the rural practice decisions of early-career physicians, particularly when these placements are of extended duration, well-structured, and accessible. Rural placements should be offered both to students from rural backgrounds, who consistently show the strongest effect, and to urban students who may develop an increased interest in rural practice when exposed to long, immersive placements. These findings support the integration of rural placements into a global policy aimed at achieving a more equitable geographic distribution of healthcare professionals. Although the effectiveness of rural placements is already recognized in international frameworks such as the World Health Organization (WHO) guidelines, their implementation remains uneven across countries.

## Background

 Disparities in access to healthcare pose a significant challenge worldwide, primarily driven by the unequal distribution of health professionals between urban and rural areas. Rural regions in many countries are facing an increasing shortage of physicians, which jeopardizes healthcare equity and exacerbates existing disparities.

 Among the strategies proposed to attract and retain young physicians in these areas, rural clinical placements have gained increasing importance. These placements provide direct immersion into the realities of rural medicine, characterized by a wide range of clinical situations, greater autonomy, a holistic approach to patient care, and strengthened ties with the local community. They often serve as a formative professional experience that can reveal or reinforce a vocation.

 The literature indicates that early exposure to rural medicine may positively influence intention to practice rurally. However, the findings are mixed. Some studies, such as the evaluation of the Parallel Rural Community Curriculum (PRCC) at Flinders University,^1^ identify prolonged rural exposure as a key determinant in the choice to establish practice in a rural setting. Others, such as Leaune and colleagues’ systematic review,^2^ report a progressive decline in positive attitudes toward underserved populations as medical training advances. Several variables appear to mediate this relationship: the student’s geographic or socio-economic background, their intended specialty, the quality of the placement and mentorship, and the perceived living conditions during the immersion, as highlighted in a 2019 article in *Gestions Hospitalières* on hospital attractiveness.^3^

 The impact of placements often extends beyond stated intentions, shaping students’ perceptions of rural practice, enhancing clinical competence, and reinforcing their sense of efficacy in unfamiliar environments. Despite having structured programs like the PRCC in Australia, international data remain fragmented, marked by significant variability in study contexts, evaluation criteria, and operational definitions. Unlike many previous reviews that focused primarily on students’ stated intentions, our study synthesizes data on actual rural practice outcomes, providing stronger evidence for workforce planning.

 In this context, a comprehensive review of the literature is necessary. The primary objective of this systematic review was to evaluate the effect of rural placements on the actual establishment of practice in rural areas. The analysis also examines several factors that may affect the decision to practice in a rural setting, including the duration of practice, intentions regarding practice location, students’ rural backgrounds, satisfaction with the placement, and its duration and structure.

## Methods

###  Study Types

 We conducted a systematic review that included both quantitative and qualitative studies assessing the impact of rural placements on the professional location choices of medical students. The selected studies primarily consisted of retrospective or prospective cohort studies, cross-sectional studies, randomized controlled trials, and qualitative studies focusing on students’ perceptions and motivations. No case-control studies were included, and none were identified among the full-text articles assessed for eligibility.

###  Target Population

 The participants included medical students or residents who had completed a clinical placement in a rural area. There were no restrictions regarding age, gender, ethnicity, or specialty.

###  Interventions

 The evaluated interventions comprised clinical placements or training programs specifically conducted in rural settings, involving substantial immersion and structured supervision.

###  Comparators

 Comparator groups consisted of students who had not participated in a rural placement or had completed a placement in an urban area.

###  Outcome Measures

 The primary outcome was the actual establishment of medical practice in a rural area following training. This measure allowed us to objectively assess the impact of rural placements on the subsequent geographic distribution of healthcare professionals.

 Additional variables analyzed included the duration of rural practice, the impact of a rural background, placement length, intended practice location (pre- and post-placement), placement satisfaction (teaching quality, supervision, learning environment), and broader perceptions related to the rural experience (attitudes toward rural practice, perceived attractiveness, and rural living conditions).

 Although related, these constructs were considered distinct.

###  Language and Publication Period

 Articles published in English or French until June 2024 were included, with no restrictions on the original publication date.

###  Literature Search Strategy

**Figure 1:**
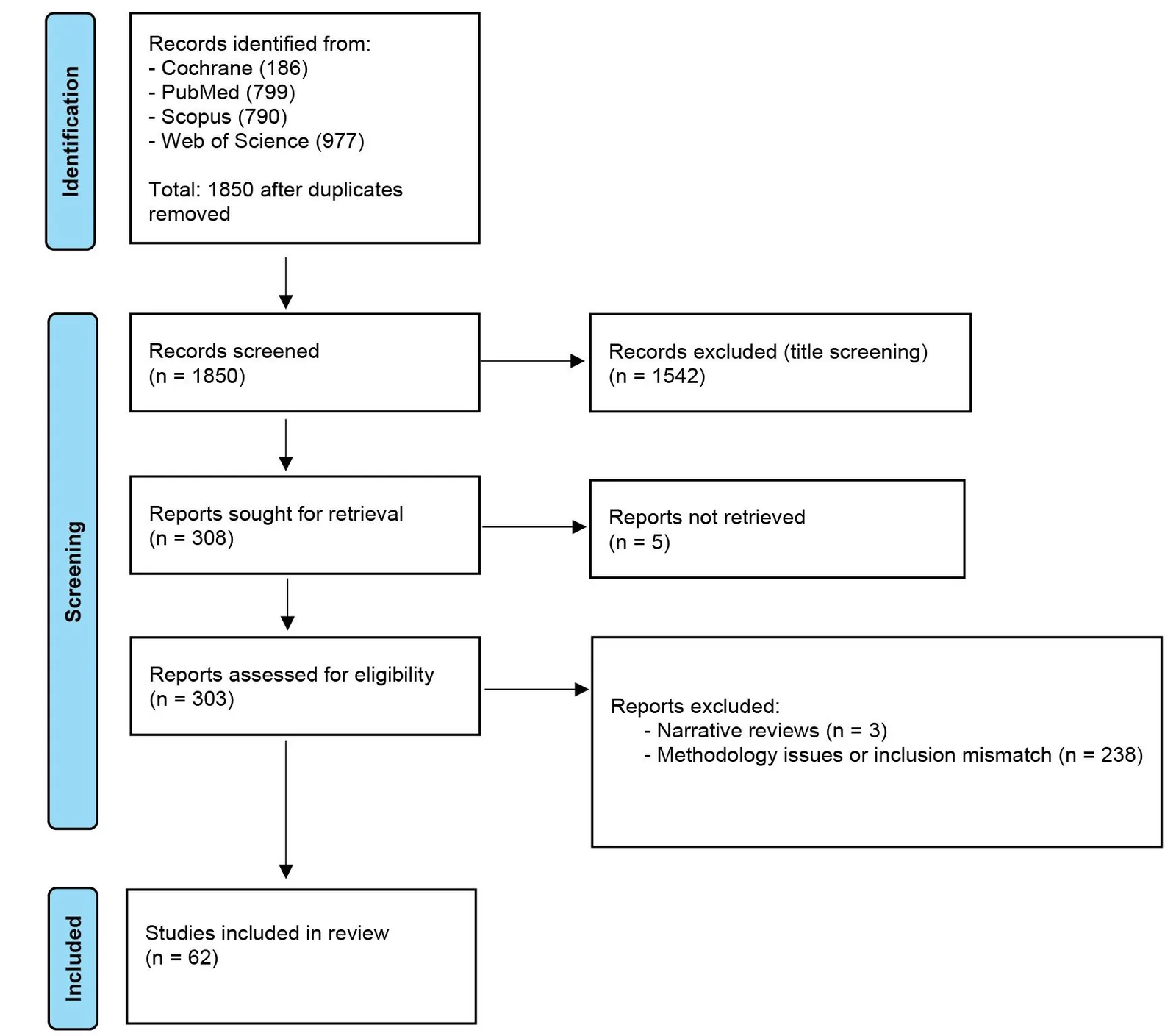
Study Selection Diagram.

 A systematic search was conducted in the following databases: PubMed, Embase, Web of Science, and the Cochrane Library. The search strategy combined keywords and MeSH terms related to the following themes: “medically-underserved area,” “medical student,” “healthcare education,” “rural career intention,” “career choice,” and “clinical placement,” using Boolean operators AND/OR (See Supplementary file 1). Two independent reviewers carried out article selection and abstract screening. They performed dual screening of titles, abstracts, and full texts based on predefined inclusion criteria. References were imported into the Rayyan software, and duplicates were removed prior to selection. Studies excluded for “inclusion mismatch” did not meet the predefined inclusion criteria (eg, wrong population, absence of a rural placement, or lack of rural practice outcomes), while studies excluded for “methodology issues” lacked extractable data, had unclear methods, or presented limitations preventing meaningful analysis (See Figure 1).

 One German study identified during full-text screening was excluded because it assessed attitudes toward rural practice using a voluntary before–after design without reporting career outcomes or comparable measures of rural career intention. It therefore did not meet our predefined eligibility criteria and could not be included in the qualitative synthesis or the meta-analysis.

###  Data Extraction, Risk of Bias Assessment, and PRISMA Framework

 Data were extracted and entered into a table detailing the characteristics of the included studies^5-66^ (Supplementary file 2), which incorporated study characteristics, the population studied, specifics of the rural placement, and the main results. The risk of bias was assessed using the RoB 2.0 tool for randomized controlled trials and the ROBINS-I tool for non-randomized studies. The full study protocol was registered in PROSPERO^4^ (registration number: CRD420251025559), and the study adhered to PRISMA (Preferred Reporting Items for Systematic reviews and Meta-Analyses) guidelines for systematic reviews.

###  Data Synthesis

 A descriptive analysis was conducted for all studies by 2 reviewers. Where possible, studies were included in the meta-analysis when the relative risk (RR) could be calculated.

 RRs were calculated only when studies reported sufficient data to construct a 2×2 table, including the number of students who completed a rural placement and subsequently practiced in a rural location, compared with those who did not complete a rural placement. When these four values could not be extracted or inferred from the article, RR calculation was not possible and the study was included in the qualitative synthesis only.

 For example, in McGrail et al, we extracted the number of participants who spent more than 40% of their postgraduate training in a rural area (“exposed” group) and the number who were practising rurally at the time of the survey. The study reported that 214 out of 275 exposed participants (78.4%) were practising rurally, compared with 87 out of 841 participants (10.4%) in the non-exposed group. These raw counts were used to construct the 2×2 table and calculate the relative risk (RR = 7.55). This illustrates exactly how events and totals were derived for inclusion in the meta-analysis.

 A random-effects meta-analysis was performed using R software (version 4.3.3) and the Meta package. Heterogeneity was assessed using I^2^ and τ^2^ statistics.

###  Definitions

 In sections where the term “rural” is used without referring to a specific study, it is used in a general and descriptive sense, meaning any non-urban or underserved area. This generic use does not impose a uniform definition, and each study’s specific definition was kept for quantitative analyses.

 The operational definition of “rural” varied substantially across studies and generally followed national classification systems. In Australian studies, rurality was most often defined using government remoteness classifications such as the Australian Standard Geographical/Statistical Geography Standard – Remoteness Areas (ASGC/ASGS–RA; rural = RA2–RA5)^6,9,11,18,23,28,31,34-36,39,42,55,61,63^ the Modified Monash Model (MMM; rural = MM2–MM7 or MM3–MM7)^5,8,14,17,22,43,46,52,54,55,60-62^ or, in earlier work, the Rural, Remote and Metropolitan Areas (RRMA; rural = RRMA3–5, remote = RRMA6–7).^20,50,59,64^ In the United States, studies mainly relied on Rural–Urban Commuting Area (RUCA) codes (rural = RUCA ≥4),^7,16,21,37^ Office of Management and Budget^12^ metropolitan versus non-metropolitan status, county-level population density categories distinguishing rural and frontier counties, or practice in medically underserved areas (eg, MUA/Health Professional Shortage Area).^38,45,49,66^ Canadian studies classified rural locations as practice outside a Census Metropolitan Area or Agglomeration,^10^ outside the provincial capital (eg, outside Winnipeg),^15^ or in communities with fewer than 10 000 inhabitants.^32,65^ New Zealand studies used the Statistics New Zealand urban–rural classification, where Rural 1–2 areas are considered rural and Principal or Secondary Urban areas are considered urban.^56-58^ Other countries applied national policy definitions of remote or rural districts (eg, community hospitals in rural districts in Thailand,^19^ remote/frontier districts in Indonesia^44^), bespoke criteria combining population size and physician density (Japan),^13^ or the very sparsely populated northern county of Finnmark in Norway.^30,33^ We did not impose a single operational definition of rurality; each study’s definition was retained for the primary analyses.

 “Early rural exposure” was also defined heterogeneously. In most studies it corresponded to a rural background, such as growing up or completing several years of secondary schooling in a rural community, or having lived mainly outside a capital city. In a smaller number of studies, early exposure referred to pre-clinical or early-clinical short placements or outreach programs in rural communities.^28,46,57^ One study used the term in a different sense, referring to early-career rural work after graduation rather than undergraduate exposure.^58^

## Results

###  Included Studies

**Figure 2:**
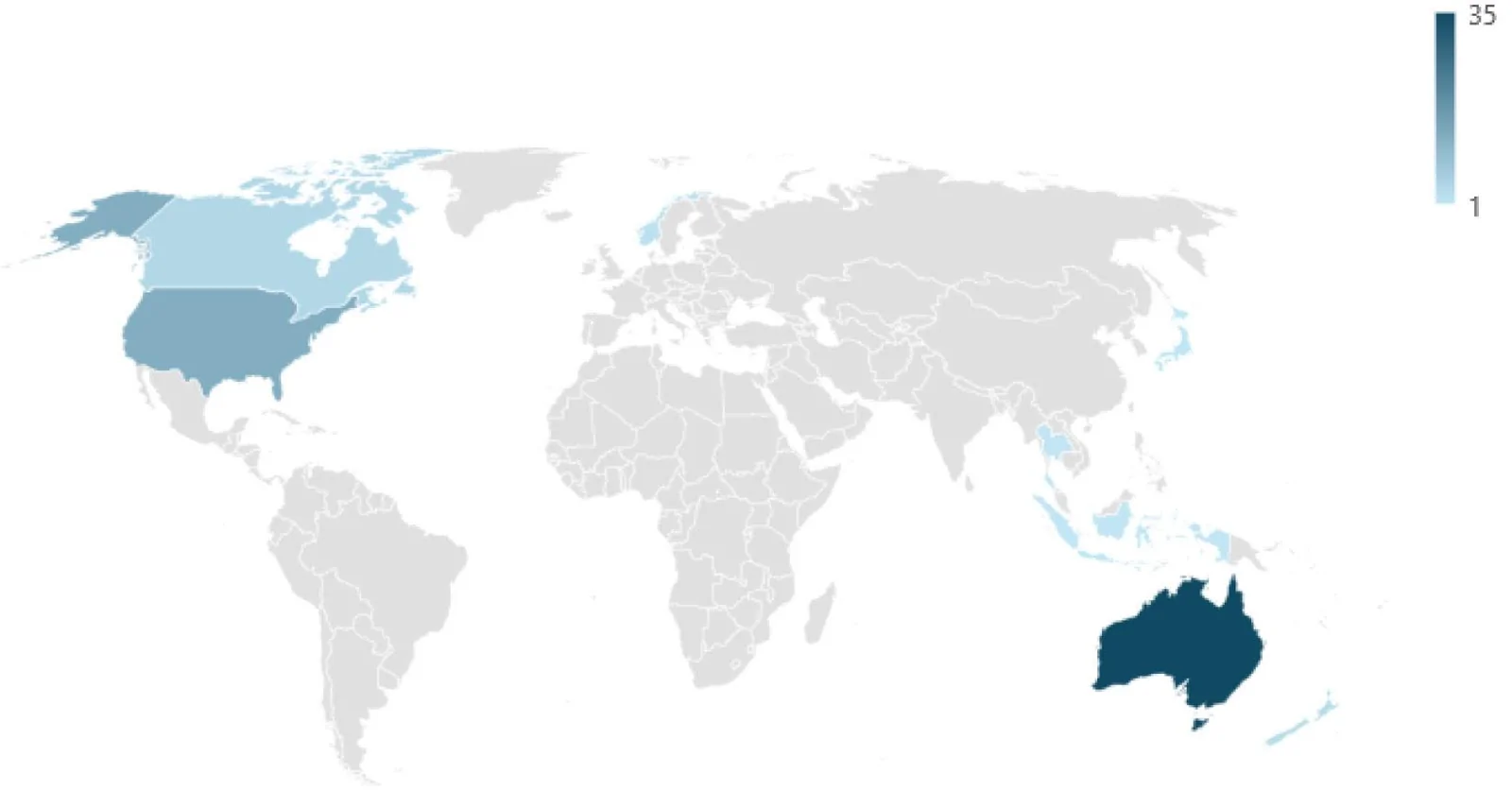
Geographical Repartition of Studies (2000–2024). Legend: Australia (n = 35), the United States (n = 13), Canada (n = 4), New Zealand (n = 3), Norway (n = 2), Japan (n = 1), Thailand (n = 1), Indonesia (n = 1), and international studies (n = 2).

 The literature search identified 1850 references. After removing duplicates and screening titles and abstracts, 62 studies^5-66^ underwent full-text review. Ultimately, 28 studies^5-8,10,11,13,14,16-19,21,23,26,27,36,43,46,49,52,54-56,58,61-63^ were included in the qualitative analysis and meta-analysis. The selection process is illustrated in the study selection diagram based on PRISMA guidelines. The geographical distribution of studies published between 2000 and 2024 is presented in Figure 2.

###  Effect of Rural Placements on Rural Practice

 Of the studies included, 50^5-19,21-23,26-28,30-39,41-44,46,48,49,51-56,58-63,66^ provided data on rural practice rates following a placement (Supplementary file 2).

**Figure 3:**
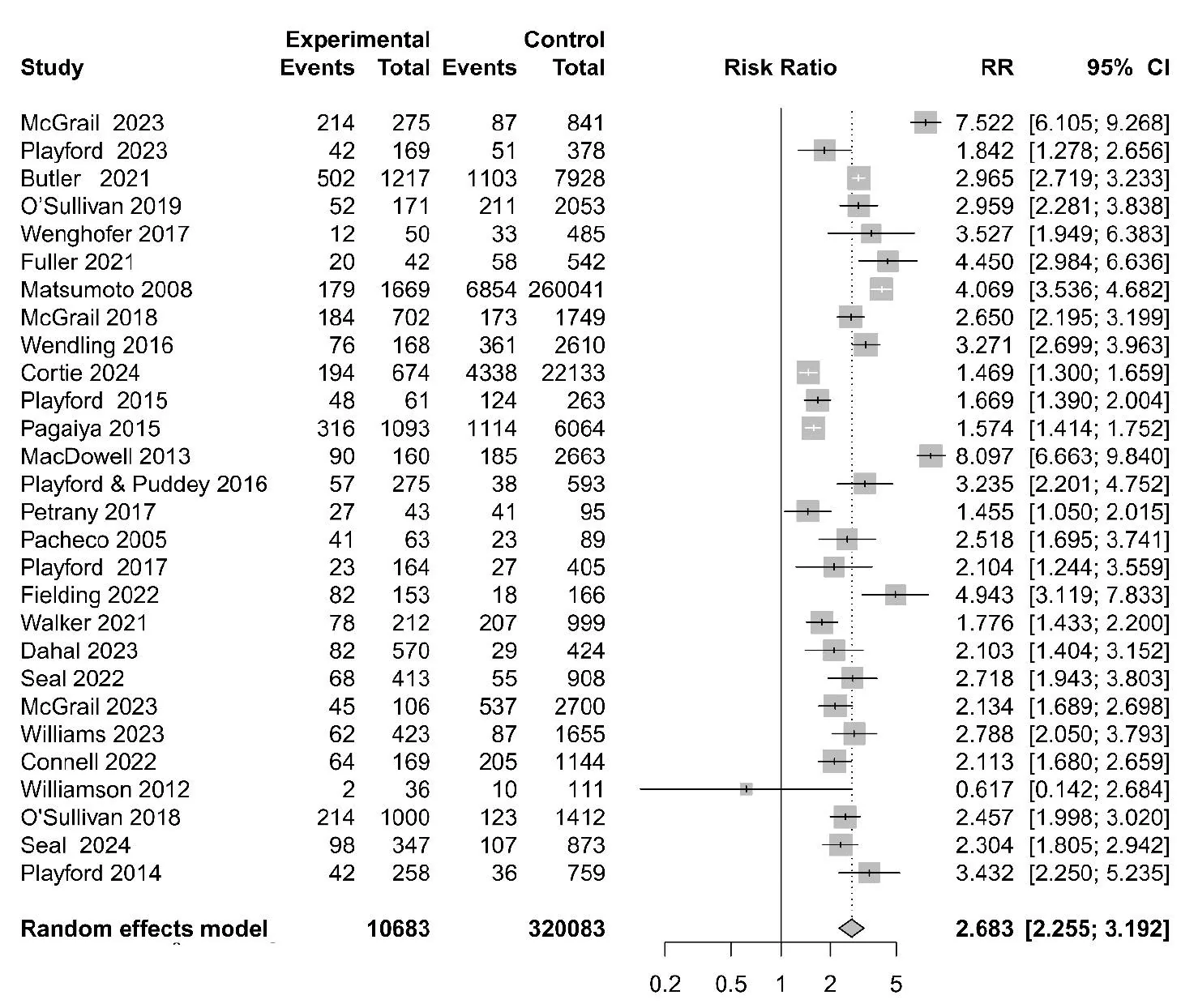
Meta-analysis Results – Forest Plot of the Effect of Rural Clinical Placement. Abbreviations: RR, relative risk; CI, confidence interval. Legend: The “events” and “totals” displayed in the forest plot were extracted directly from the raw data provided in each included study. “Events” correspond to the number of participants who both completed a rural placement or rural immersion period and subsequently practised in a rural area (exposed group), or the number of non-exposed participants who practised in a rural area (control group). “Totals” represent the full number of participants in each exposure group.

 The meta-analysis of 28 studies involving 330 766 students and early-career physicians confirmed a significant overall effect (Figure 3). The pooled RR of rural practice was 2.683 (95% CI [confidence interval]: 2.255–3.192, *P*< .0001). The heterogeneity of the results (I^2^ = 94.9%, 95% CI: 93.6–96.0%, *τ*^2^ = 0.191, 95% CI: 0.112–0.399) justified the use of a random-effects model and reflected the diversity of methodologies, populations, and geographic contexts (including in the definition of “rural”).

 Several studies reported a significant and heterogeneous effect size. For example, McGrail et al^5^ found that general practitioners (GPs) with extended postgraduate training in a rural setting had an odds ratio (OR) of 45 (95% CI: 24–84), compared to 11 (95% CI: 5–22) for specialists. Similarly, Woolley et al^22^ reported an OR of 54.6 (95% CI: 4.4–678.1) for interns who had completed their placement in a highly remote hospital (MMM6–7 areas).

 Long immersive programs confirmed this trend. In the Rockford Rural Medical Education program (United States), 56.3% of graduates were practicing in rural areas, compared to 6.9% of non-participants,^21^ OR = 17.2 (95% CI: 12.18–24.35). In Australia, the Rural Clinical School of Western Australia reported a rural practice rate of 78.7% among participants, compared to 45.5% among non-participants (OR = 4.42).^18^ Other programs such as the Rural Physician Associate Program^7^ also showed a significant difference: a 41.2% rural practice rate among participants, compared to 13.9% among non-participants.

 The Northern Ontario School of Medicine^10^ illustrated the importance of a structured rural training program, with 25.4% of its graduates establishing practice in rural areas, compared to 10.3% of graduates from other schools (OR = 2.57; 95% CI: 1.21–5.44). Similarly, Halaas et al^12^ reported that 49.7% of former students were practicing in rural areas, while 14% split their practice between rural and urban settings.

 Other studies confirmed these differences in various contexts: Pacheco et al^27^ reported a rural practice rate of 65.1% among graduates from rural residency programs, compared to 25.8% for urban-track graduates. Wendling et al^16^ found that 45% of graduates from the Rural Physician Program were practicing in rural areas, compared to 14% of other graduates.

 Even in studies reporting more moderate rates, the observed effect on rural practice remained significant. Fuller et al^11^ reported an overall rate of 16.2%. Eley et al^9^ found a rate of 40%, and Rourke et al^32^ observed that 26.9% of physicians trained at MUA were practicing in rural areas two years after completing residency, compared to a national average of 13.3%.

 Finally, McGrail et al^54^ highlighted the effect of an extended 12-week rural placement, noting that 42.5% of participants later established practice in a rural or regional area, compared to 19.9% among non-participants (OR = 2.2; 95% CI: 1.1–4.6). The effect was particularly strong among students from rural backgrounds (OR = 4.9), but also significant among those from urban backgrounds (OR = 2.8).

 Across all 50 studies—conducted in various contexts and at different stages of medical education (undergraduate, rural clinical placement, or residency)—35 studies^5-8,10,13,15-19,21-23,26-28,30,36,37,39,42-44,46,49,51-56,61,63,66^ demonstrated a significant association between participation in a rural placement and a higher likelihood of subsequently establishing practice in a rural area. In contrast, 12 studies^9,11,12,14,32-35,38,59,60,62^ did not include statistical analyses that allowed for direct measurement of this effect, and 3 studies^41,56,58^ reported non-significant results (Supplementary file 2).

###  Placement Duration

 The analysis of 21 studies^5,8,11-14,20,22,24,34,39,41,42,48,51,53,54,57,59,61,62^ provided data on the impact of placement duration.

 Extended placements—especially those lasting over one year—were significantly associated with a higher likelihood of establishing practice in a rural area after training. This dose–response relationship appeared across several cohorts and varied geographical contexts. Among the most robust data, McGrail and O’Sullivan^42^ reported a much stronger effect for placements longer than one year (relative risk ratio [RRR] = 5.22; 95% CI: 3.95–6.89) compared to placements lasting 3 to 12 months (RRR = 1.42; 95% CI: 1.08–1.88), with no significant effect noted for placements shorter than 12 weeks. This relationship was confirmed by O’Sullivan et al,^61^ who noted a progressive increase in the likelihood of rural practice based on the length of immersion: OR = 1.79 (95% CI: 1.15–2.79) for one year, OR = 2.26 (95% CI: 1.54–3.32) for 1–2 years, and OR = 4.43 (95% CI: 3.03–6.47) for more than two years.

 Several studies also emphasized the impact of extended placements on practice in the same region where the placement took place. McGrail et al^14^ reported a RRR of 3.38 (95% CI: 1.91–5.98) in favor of students who completed an 18- to 24-month placement compared to those who undertook a shorter placement (12 months). Similarly, McGrail et al^54^ reported an OR of 8.6 (95% CI: 4.5–16.3) for students who completed an extended program combined with two years of regional training in these areas.

 Comparable data were reported by Fuller et al,^11^ where the combination of one year in a rural longitudinal integrated clerkship and one year in a regional hospital was linked to subsequent rural practice (OR = 7.09; 95% CI: 3.54–14.19), compared to no rural placement.

 The positive effect of extended placements was also reflected in the stated intention rates. For example, Isaac et al^24^ observed that 91.7% of students who had completed three years of training in a Rural Clinical School expressed an intention to practice in a rural area, compared to 60% after one year (*P*= .003). Likewise, Abid et al^57^ indicated that students who had completed a rural placement of more than 33 weeks were 6.4 times more likely to declare this intention (RR = 6.4; *P*< .001) compared to those who had completed a short five-week placement.

 Longitudinal integrated clerkships, particularly those lasting at least seven months, were also associated with significantly positive effects. Crampton et al^48^ demonstrated that these clerkships promoted stronger community integration and had a notable impact on career choices. Young et al^20^ also observed a cumulative/dose–response effect: the intention to practice in a rural area increased from 9% after two weeks of exposure to 19% after four years of repeated placements.

 Some studies reported effects with ORs greater than 5: Woolley et al^22^ found an OR of 8.9 (95% CI: 1.6–50.8) for placements longer than 20 weeks, while Woolley et al^39^ reported an OR of 17.0 (95% CI: 6.9–42.1) for students who had trained in the rural generalist pathway.

 Conversely, data from McGrail et al^5^ showed that a short placement (3–12 months) was associated with an OR of 0.72 (95% CI: 0.42–1.27), while an extended placement (>1 year) was associated with an OR of 1.37 (95% CI: 0.62–3.03), with no statistical significance.

 Of the 21 studies analyzed, 13 studies^8,11,14,22,24,34,39,41,42,54,57,59,61^ demonstrated a statistically significant association between completing an extended rural placement and subsequent rural practice.

 However, six studies^12,13,20,48,51,53^ did not include statistical analyses that allowed for direct measurement of this effect, and two studies^5,62^ reported non-significant results.

 Rural practice following an extended placement was further strengthened by factors such as community integration, the immersive nature of the placements, and their repetition throughout the training program. These findings support the integration of extended, well-structured rural placements into medical education to promote sustainable practice in underserved areas.

###  Student Satisfaction

 A total of 13 studies^9,12,20,24,25,34,47,48,53,58-60,64^ included in this review addressed student satisfaction. In the study by Eley et al,^9^ 70% of students (65 out of 115) stated that their placement in a rural clinical school had strengthened their intention to practice in a rural area, and 92% stated they would make the same choice again if given the opportunity. Similarly, Young et al^20^ found that 85% of students perceived a positive impact of the program on their rural career aspirations, with an overall satisfaction rate of 87%, including both clinical and social aspects of the placement.

 In the study by Walker et al,^25^ 85% of students expressed an increased interest in rural practice following their rural clinical school experience, and 86% considered rural practice as a career option. Isaac et al^24^ highlighted a shift in intentions using an interest rating scale: among students already inclined toward rural practice, the score rose from 3.8 to 4.1 out of 5 (*P*= .02); conversely, it significantly decreased among those initially drawn to urban practice (*P*= .002). Furthermore, Moffatt^47^ found that 64% of surveyed students (*n* = 1020) felt their rural rotation strengthened their intention to practice in a rural area, with 46% describing this effect as “very strong.”

 The impact of the placement varied depending on the context. In the study by Williamson et al,^58^ a positive influence was reported by 56% of former students from Dunedin, compared to 24% in Christchurch and 15% in Wellington. Eley et al^59^ also identified a correlation between placement duration and satisfaction level: 96% of students said they would choose the same placement again, with a mean satisfaction score of 4.51/5. Those who had spent two years in the program were significantly more enthusiastic than those who had stayed for only one year (*P*= .003).

 The findings of Williams et al^60^ confirmed these trends: 91.7% of students felt well supported, 87.8% reported a positive impact on their well-being, and 90.4% expressed an increased interest in a rural career after the placement. Eley and Baker^64^ likewise noted high satisfaction (4.06/5) and a strengthened interest in rural medicine (3.74/5).

 In addition to quantitative data, qualitative studies helped to clarify the underlying psychological mechanisms. Halaas et al^12^ emphasized the importance of placements (nine months) in fostering lasting relationships with patients and teams, enhancing feelings of competence and usefulness, and supporting integration into the local community. Crampton et al^48^ also reported positive psychological effects: a sense of usefulness, increased confidence, and improved self-esteem. However, their study also noted potential limitations such as social isolation, lack of clear educational objectives, and difficulties with accommodation.

 Overall, 2 of the 13 studies^24,59^ reported a significant effect on satisfaction with rural placement programs. In contrast, 11 studies^9,12,20,25,34,47,48,53,58,60,64^ did not include statistical analyses that allowed for direct measurement of this effect.

 The results highlight a correlation between the quality of the rural placement experience and an increased interest in a rural career—both motivationally and psychologically. These effects were particularly strong in extended placements that were well-supervised and provided genuine immersion in the local community.

###  Impact of Rural Background

 The analysis also examined how students’ rural backgrounds influenced their decision to practice in a rural area. A total of 46 studies^5,6,8,9,11-18,22-25,28,30-32,34-37,41-45,48,50-63,65,66^ provided data on this variable.

 Williams et al^55^ reported an OR of 8.29 (95% CI: 4.22–16.26), while Tate and Aoki^15^ reported an OR of 8.37 (95% CI: 6.45–10.84), and Putri et al^44^ found an OR of 19.9 (95% CI: 12.3–32.3) for students from remote rural areas.

 The impact of rural background was amplified when combined with an extended rural placement. McGrail and O’Sullivan^42^ reported a RRR of 17.4 (95% CI: 11.6–26.1) among physicians from rural backgrounds who had completed over a year of training in the same region. Other studies confirmed this synergy: Playford et al^6^ reported an OR of 5.00 (95% CI: 2.36–13.3), and Seal et al^52^ reported a RR of 4.8 (95% CI: 3.1–7.5).

 A meta-analysis of ten studies conducted by Ogden et al^51^ also concluded that a rural background had a significant overall effect on rural practice, with an OR of 2.71 (95% CI: 2.12–3.46).

 Finally, Johnson et al,^53^ who analyzed 25 studies, noted that in some cases, a rural background was an even stronger predictor of rural practice than having completed a rural placement.

 In total, 33^5,6,8,11,13-16,22-23,25,28,30,36,41-44,48,50-57,59-62,65,66^ of the 46 studies reported a statistically significant association between rural background and subsequent rural practice. In contrast, eight studies^9,12,17,24,31-35,37,58^ did not include statistical analyses that allowed for direct measurement of this effect, and four^12,18,45,63^ reported non-significant results.

###  Intention to Establish Practice in a Rural Area

 A total of 10 studies^20,24,25,29,41,45,47,50,58,64^ included in this review examined the intention to establish practice in a rural area following a rural placement.

 In this review, career intention was treated as a multidimensional construct, and we distinguished pre-existing rural intent from the change in intent occurring during or after the placement.

 The intention of medical students to establish practice in a rural area constituted a key predictor of actual rural practice after training. Many of the studies included in this review examined how this intention evolved following a rural placement.

 Several studies reported a significant increase in rural intent after a placement, especially when it was extended or repeated. In the Australian JFPP program, Young et al^20^ found that the intention to become a rural GP applied to 9% of students after a single placement, 19% after four placements, and reached 65% among those who completed the entire program. This result illustrated a clear dose–response relationship: the more exposure students had, the stronger their inclination toward rural practice.

 The stability of this intention was also highlighted. Isaac et al^24^ demonstrated that only 6.3% of students who initially expressed rural intent later changed their preference toward urban practice after one year, indicating a strong persistence of their initial motivation.

 Beyond the duration or frequency of placements, the nature of the clinical experience also appeared to play a determining role. Moffatt^47^ reported that students who had substantial clinical responsibility during their placement were significantly more likely to feel positively influenced in their intention to practice in a rural area (OR = 3.55; 95% CI: 2.05–6.14). Similarly, the autonomous management of routine cases was also associated with stronger rural intent (OR = 2.16; 95% CI: 1.43–3.28).

 Jones et al^50^ showed that placements completed in very remote areas significantly increased the likelihood of rural intent (OR = 1.77; 95% CI: 1.25–2.49), while late-stage urban placements had the opposite effect (OR = 0.47; 95% CI: 0.32–0.69).

 Additional results from Taylor and Goletz^45^ confirmed this trend: 21.6% of students who completed a rural placement expressed an intent to practice rurally, compared to 12.3% of those trained only in urban settings (*P*= .01).

 In total, 6 of the 10 studies^20,24,41,45,47,50^ reported a statistically significant effect on students’ rural intent after a placement in a rural area. Two studies^29,58^ reported non-significant results, and two studies^25,64^ did not include statistical analyses enabling direct measurement of this effect.

 Overall, these results suggest that rural placements strengthen rural intent, particularly when the placements are extended, intensive, or well-structured. This intention can be shaped by the quality of the educational experience, the geographical context of the placement, and students’ personal motivations.

###  Sustainability of Practice

 A total of 16 studies^11-14,19,21,33,35,39,48,50,52-55,62^ included in this review examined the sustainability of rural practice following training. Findings varied depending on context, specialty, and practitioner profile.

 Halaas et al^12^ reported that between 48% and 49% of graduates from 1985 to 2006 had spent their entire careers in rural settings, demonstrating a remarkable level of long-term commitment. Similarly, Playford et al^35^ reported that 44% of physicians who had completed a rural placement were still practicing in a rural area ten years later.

 The average duration of rural practice was also well documented. MacDowell et al^21^ reported a mean of 5.3 years (ranging from 0.5 to 11.3 years) among participants in the Rockford Rural Medical Education program, with 68% remaining in their initial practice location. Pagaiya et al^19^ reported an average of 4.2 years for graduates of the Collaborative Project to Increase Rural Doctors program, compared to 3.4 years for graduates of standard programs, with a 20% reduction in the risk of leaving rural practice (Hazard ratio = 0.795; 95% CI: 0.73–0.86).

 Fuller et al^11^ clearly illustrated trends over time: 29.4% of practitioners worked in rural areas during their first post-graduate year (PGY1), and 20.4% continued to practice rurally in PGY8. After a decline between PGY1 and PGY4, a slight rebound was observed, suggesting a degree of long-term stability.

 The length of rural practice also seemed to be influenced by the physician’s profile. Matsumoto et al^13^ found that physicians from rural backgrounds were significantly more likely to remain in rural areas over the long term (OR = 1.90; 95% CI: 1.04–3.48), and that GPs stayed longer than specialists (OR = 32.07; 95% CI: 4.43–232.24).

 Seal et al^62^ also highlighted a high retention level: 71.4% of GPs practicing in rural areas at five years were still practicing there at PGY10, compared to 29.0% of non-GP specialists.

 In total, 7 out of 16 studies^13,19,50,52,54,55,62^ reported a significant effect of rural placement programs on the duration of rural practice. In contrast, eight studies^11,12,21,33,35,39,48,53^ did not include statistical analyses that allowed for direct measurement of this effect, and one study^10^ reported non-significant results.

###  Additional Factors Influencing Rural Practice

 In addition to direct experience with rural placements, several studies highlighted the significant impact of contextual, structural, or individual factors on the decision to establish practice in rural areas.

####  A. Service Requirement

 Among the identified levers was the requirement for rural service. Physicians with a contractual obligation were significantly more likely to establish practice in rural areas, with ORs ranging from 3.14 to 5.60 depending on their specialty.^5^ Other studies confirmed this effect, such as Tavernier et al^66^ who reported an OR of 3.1 for practitioners assigned to medically-underserved areas. Similarly, participation in a Medical Rural Bonded Scholarship tripled the likelihood of rural practice,^23^ while some financial incentive programs increased this probability by as much as 18.6 times.^44^

####  B. Choice of Specialty

 The choice of specialty also influenced practice location. GPs were significantly more likely to establish practice in rural areas (OR = 3.5),^55^ and Woolley et al^22^ reported an OR of 4.4 for GPs and up to 26.4 for specialists already practicing in these areas.

 Certain training programs further reinforced this trend. Students who completed the Rural Physician Associate Program were more likely to work in primary care and to remain in the state of Minnesota after their training.^7^ Similarly, graduates of the PRCC were 2.6 times more likely to be registered as GPs.^46^

####  C. Personal and Socio-Economic Background

 Personal and socio-economic factors also appeared to play a major role. Students from more disadvantaged backgrounds (Index of Relative Socio-EconomicAdvantage and Disadvantage indices 1–4) were more likely to choose rural practice (OR = 4.04).^23^ Furthermore, an early interest in general practice was strongly associated with rural intent (RR = 4.5).^57^ Finally, a Norwegian study^30^ found that interns who had studied abroad were more likely to establish practice in the rural regions of northern Norway (OR = 3.4).

 Overall, these results demonstrate that rural placement experience is only one component within a broader set of interrelated determinants—including financial, academic, motivational, and contextual factors—that contribute to encouraging young physicians to establish practice in a rural setting.

## Discussion

 Our results indicate that rural placements significantly influence future physicians’ choices regarding career locations. However, this inclination toward underserved areas does not arise solely from a single geographical exposure, but rather from a combination of personal, pedagogical, structural, and motivational factors.

 Placement duration, pedagogical quality, and rural immersion are frequently reported as important contextual factors influencing perceptions of rural practice. Extended, well-supervised placements may support clinical skill development and may contribute to a more positive perception of rural practice, as suggested by several studies. These experiences may facilitate community integration, which several authors describe as an influential factor when considering long-term rural practice. In contrast, short, disorganized, or poorly supported placements conducted in challenging conditions may prove counterproductive—fueling resistance rather than inspiring commitment.

 Students’ rural background emerges as a powerful lever, reflecting a pre-existing familiarity with the rural environment, a cultural rootedness, and often a sense of indebtedness to their community of origin. In such cases, the rural placement reinforces intent, solidifying a project already in the making. For students from urban backgrounds, immersion in a rural setting can serve as a revelation—sometimes challenging stereotypical representations and generating an unexpected interest in a form of practice perceived as rich, human-centered, and rewarding.

 In addition to the skills acquired, rural placements offer a formative human experience: close relationships with patients, a visible impact on community health, and a sense of social utility. These subjective yet essential elements help shape a career plan rooted in strong ethical and relational values.^12,47^

###  Limitations

 Nonetheless, these results should be interpreted with caution and present several limitations. First, the heterogeneity of study methodologies, variability in the definitions of “rural,” and predominance of retrospective designs limit the generalizability of certain conclusions. Second, the conversion of intention into actual practice depends on multiple factors beyond the placement itself: working conditions, work–life balance, job prospects, and the partner’s integration. Studies do not always include these elements as adjustment factors. Third, the varying quality of studies and potential publication bias may have influenced study selection and introduced bias into our results. However, the use of PRISMA criteria helped to mitigate this effect. The funnel plot (Supplementary file 3) shows moderate asymmetry, consistent with a possible publication bias or small-study effect.

 Fourth, most included studies were conducted in high-income countries, particularly Australia, the United States, and Canada. Evidence from low- and middle-income countries is remarkably scarce. This geographic imbalance limits the generalizability of our findings to different health-system contexts. Rural medical practice, training structures, and workforce challenges are likely to differ substantially in low- and middle-income countries, and additional research is needed to determine whether rural placements have comparable effects in these settings.

 Finally, an important limitation relates to the potential impact of self-selection bias. Students already motivated to pursue a rural career are more likely to choose optional rural placements, particularly the longer or more immersive programs. As a consequence, comparisons with standard curricula—which involve fewer students predisposed to rural practice—may inadvertently exaggerate the apparent effect of rural placements. This phenomenon may also partially explain why extended placements are more strongly associated with rural career choice. While this self-selection may play a positive reinforcing role, its magnitude remains unknown and must be acknowledged when interpreting observed associations.

###  Key Recommendations

 The implications of these results nonetheless highlight the need to conceptualize rural medical training within a systemic framework—combining early identification of interest, personalized support, and alignment with local health services and regional workforce planning strategies. The following recommendations emerge from this review:

- Target the recruitment of students from rural areas or with strong social motivation;
- Expand the availability of rural placement sites through partnerships between universities, local health centers, and peripheral hospitals, to ensure greater access for interested students;
- Develop immersive, multi-semester pathways that are pedagogically structured and tailored to the practical realities in the most underserved areas;
- Promote rural tracks within university curricula and rural clinical placement criteria;
- Implement longitudinal follow-up of former placement participants to track trajectories, better understand the drivers of rural practice, and refine retention policies;
- Link these placements to sustainable post-establishment incentives (eg, support for isolated practice, housing, flexible work options, interprofessional coordination).

## Conclusion

 Rural placements during medical school are associated with a higher likelihood of rural practice later in physicians’ careers. Their impact extends beyond simply discovering a healthcare environment: they offer an immersive experience capable of transforming perceptions, anchoring motivation, consolidating skills, and, in some cases, sparking a vocation.

 To be fully effective, these placements must meet several conditions: sufficient duration, high-quality supervision, community immersion, and integration within a coherent training pathway. They should also be integrated into a broader policy to enhance the appeal of rural practice—linking training, incentives, tailored support, and rural career recognition.

 Strengthening these programs, making them accessible to a diverse range of student profiles, and positioning them not as peripheral options but as central components of medical education, offers a concrete path toward addressing the persistent challenges of medical workforce distribution and territorial equity.

 This study has helped to clarify the mechanisms influencing young physicians’ practice location decisions, beyond financial incentives or administrative constraints. It highlights the importance of local integration, recognition of rural pathways, and the decisive role that initial training can play in reducing inequalities in access to healthcare.

 While the effectiveness of rural placements has long been acknowledged by major international bodies, including the World Health Organization (WHO) recommendations on rural recruitment and retention, our review shows that translating these guidelines into practice remains inconsistent across regions. Strengthening the adoption of these evidence-based strategies is therefore essential to reduce geographic disparities in access to care.

## Acknowledgements

 The authors would like to thank Fiona Ecarnot, PhD, Université Marie & Louis Pasteur, Besançon, France, for editorial assistance.

## Disclosure of artificial intelligence (AI) use

 Not applicable.

## Ethical issues

 Not applicable.

## Conflicts of interest

 Authors declare that they have no conflicts of interest.

## Supplementary files


Supplementary file 1. Search Equation.

Supplementary file 2. Detailed Characteristics of Included Studies Investigating Rural Training And Physician Workforce Outcomes.

Supplementary file 3. Funnel Plot for Evaluating Publication Bias.
