# Supplementary material for: The Impact of Rural Clinical Placements on Medical Students’ Career Choices: A Systematic Review
Source: Int J Health Policy Manag. 2026 May 5;15:9328. doi: 10.34172/ijhpm.9328 (PMC13498956; doi:10.34172/ijhpm.9328)
Supplement: Supplementary file 1 [file ijhpm-15-9328-s001.pdf]

Article title: The Impact of Rural Clinical Placements on Medical Students' Career Choices: A Systematic Review

Journal name: International Journal of Health Policy and Management (IJHPM)

Authors' information: Marc-Antoine Schirm[1], Jan Chrusciel[2], Maxime Thorigny[2], Aline Hurtaud[3], Nathalie Bednarek[4], Stephane Sanchez[1,5]*

[1]Public Health Department, Champagne Sud Hospital, Troyes Hospital, Troyes, France.

[2]Department of Neonatology, Reims University Hospital Alix de Champagne, Reims, France.

[3]Department of General Practice, Faculty of Medicine, University of Reims Champagne-Ardenne, Reims, France.

[4]Laboratory REGARDS, Research Unit 6192, University of Reims Champagne-Ardenne, Reims, France.

[5]Research Unit 3797 VieFra, Faculty of Medicine, University of Reims Champagne-Ardenne, Reims, France.

***Correspondence to:** Stephane Sanchez; Email: stephane.sanchez@hcs-sante.fr

Citation: Schirm MA, Chrusciel J, Thorigny M, Hurtau A, Bednarek N, Sanchez S. The impact of rural clinical placements on medical students' career choices: a systematic review. Int J Health Policy Manag. 2026;15:9328. doi:10.34172/ijhpm.9328

Supplementary file 1. Search Equation

("education, medical"[Mesh terms] OR "medical education" [Title/Abstract] OR "healthcare education" [Title/Abstract] OR "clinical education" [Title/Abstract] OR "clinical placement*" [Title/Abstract] OR "clinical training" [Title/Abstract] OR "residency education" [Title/Abstract] OR "students, medical"[MeSH Terms] OR "medical student*" [Title/Abstract]) AND ("medically underserved area"[MeSH Terms] OR "underserved region*" [Title/Abstract] OR "rural health services"[MeSH Terms] OR "rural population"[MeSH Terms] OR "rural population"[Title/Abstract] OR "rural communities" [Title/Abstract] OR "rural residents" [Title/Abstract] OR "rural health services"[Title/Abstract] OR "medically underserved area"[Title/Abstract]) AND ("intention"[MeSH Terms] OR "career choice"[MeSH Terms] OR "intention"[Title/Abstract] OR "career choice"[Title/Abstract] OR "rural career intention*" [Title/Abstract]
