# Supplementary material for: The Impact of Rural Clinical Placements on Medical Students’ Career Choices: A Systematic Review
Source: Int J Health Policy Manag. 2026 May 5;15:9328. doi: 10.34172/ijhpm.9328 (PMC13498956; doi:10.34172/ijhpm.9328)
Supplement: Supplementary file 2 [file ijhpm-15-9328-s002.pdf]

Article title: The Impact of Rural Clinical Placements on Medical Students' Career Choices: A Systematic Review

Journal name: International Journal of Health Policy and Management (IJHPM)

Authors' information: Marc-Antoine Schirm[1], Jan Chrusciel[2], Maxime Thorigny[2], Aline Hurtaud[3], Nathalie Bednarek[4], Stephane Sanchez[1,5]*

[1]Public Health Department, Champagne Sud Hospital, Troyes Hospital, Troyes, France.

[2]Department of Neonatology, Reims University Hospital Alix de Champagne, Reims, France.

[3]Department of General Practice, Faculty of Medicine, University of Reims Champagne-Ardenne, Reims, France.

[4]Laboratory REGARDS, Research Unit 6192, University of Reims Champagne-Ardenne, Reims, France.

[5]Research Unit 3797 VieFra, Faculty of Medicine, University of Reims Champagne-Ardenne, Reims, France.

***Correspondence to:** Stephane Sanchez; Email: stephane.sanchez@hcs-sante.fr

Citation: Schirm MA, Chrusciel J, Thorigny M, Hurtau A, Bednarek N, Sanchez S. The impact of rural clinical placements on medical students' career choices: a systematic review. Int J Health Policy Manag. 2026;15:9328. doi:10.34172/ijhpm.9328

Supplementary file 2. Detailed Characteristics of Included Studies Investigating Rural Training and Physician Workforce Outcomes.

Reference	Year	Country	Study Type	Study Population	Number of Participants	Intervention	Follow-Up Duration	Main Findings	Factors Influencing Career Choices	Level of Evidence
McGrail et al[5]	2023	Australia	Retrospective cohort study based on two surveys (MABEL and UQMediCoS)	Australian doctors graduated between 2000 and 2018	1116	1-to 2-year postgraduate rural immersion	Up to 10 years post-graduation	Post-graduate rural training time associated with an increase in practising rurally; OR = 45; 95% CI (24 – 84) for GPs	Rural background, rural immersion during studies, postgraduate rural training.	2b
Playford et al.[6]	2023	Australia	Longitudinal cohort study	Medical graduates between 2010 and 2016	547	1-year initial intention and rural immersion training	Follow-up from medical school entry to workplace location between 2014 and 2019 (approx. 8 to 13 years depending on cohorts)	Students with persistent rural intention 7 times more likely to practising rurally OR = 6.76; 95% CI (2.98 – 15.33)	Rural origin, rural immersion (RCSWA), rural intention at the end of studies, interaction between these elements; end-of-studies intention is the best predictor of actual rural practice.	1b

Butler et al.[7]	2021	USA	Retrospective observational comparative study (RPAP vs nonRPAP)	University of Minnesota Medical graduates 1975–2017	9 145	Rural Physician Associate Program (RPAP) – 3rd-year 9-month rural immersion	Up to 42 years, cohorts followed 1975 to 2017	41.2% of RPAP graduates enter rural practice compared to only 13.9% of nonparticipants.	Participation in a rural placement (RPAP), family medicine training, prolonged community exposure, selfselection of interested students .	2b
O'Sullivan et al.[8]	2019	Australia	Longitudinal cohort study with multivariate analysis	Monash University medical graduates 2008–2016	2 224	Extended Rural Cohort (ERC) programme 2- to 3-year rural immersion	1 to 9 years postgraduation (2017 workforce data)	Students who ranked ERC as their last choice (3rd) had an increased probability of settling in a rural area after training (OR: 3.27). This suggests that prolonged rural exposure during training has a structuring effect, even among those without an initial strong rural intention.<br><br>ERC – 1st preference: OR: 2.69 (95% CI: 1.47–4.92)<br><br>ERC – 2nd preference: OR: 2.94 (95% CI: 1.47–5.87)	Participation in a rural programme from medical school entry (ERC), prolonged duration of rural exposure, rural origin, self-selection of students interested in rural practice.	2b
								ERC – 3rd preference: OR: 3.27 (95% CI: 1.57–6.81)		
Eley et al.[9]	2012	Australia	Mixed-methods longitudinal study	Rural Clinical School medical graduates 2002–2010	115	1- to 2-year Rural Clinical School – rural immersion	Up to nine years post-graduation for the 2002.	40% of RCS graduates work in a rural area (compared to 13.9% of the general population). Strong influence of prolonged rural exposure.	Rural origin, prolonged exposure during rural placements (RCS), family/personal constraints, quality of mentors during the placement.	2b

Wenghofer et al.[10]	2017	Canada	Retrospective cross-sectional observational study	Family medicine doctors graduated in Ontario 2009 onwards	535	Undergraduate (UG) or postgraduate (PG) NOSM rural immersion programme	Data collected in 2013 for cohorts graduated from 2009 onwards	25.4% of graduates with NOSM training practice in rural Ontario, compared to 10.3% for non-NOSM graduates. UG medical education at NOSM is associated with a higher probability of rural practice: OR: 2.57 (95% CI: 1.21–5.44), P = .014	Training at Northern Ontario School of Medicine (NOSM), particularly at the PG level, age at graduation, geographical origin (North vs South).	2b
Fuller et al.[11]	2021	Australia	Observational cross-sectional study	Deakin University graduates 2011–2018	584	1- to 2-year Rural Longitudinal Integrated Clerkship (LIC) and rural hospital immersion	Up to 8 years post-graduation	A 1-year rural clerkship (LIC) in Year 3 plus a 1-year placement in a rural regional hospital in Year 4 increases the probability of rural practice sevenfold; OR: 7.09 (95% CI: 3.54–14.19)	Rural origin, participation in a long-term rural immersion programme (LIC), two consecutive years of rural clinical placement, rural service obligations (BMP/MRBS), and targeted student selection.	2b
Wagstrom Halaas et al.[12]	2008	USA	Retrospective descriptive observational study	Rural Physician Associate Program (RPAP) graduates 1971–2007	901	9-month RPAP rural immersion in Year 3	Up to 36 years post-participation (longitudinal follow-up via database updated every 3 years)	49.7% of RPAP graduates work in a rural area compared to 13.9% of the general population; 58% of currently practicing RPAP graduates have spent more than 50% of their career in a rural setting.	Participation in the RPAP programme, rural origin, interest in rural medicine, prolonged rural exposure.	2b
Matsumoto et al.[13]	2008	Japan	Retrospective cohort study	Jichi Medical University (JMU) graduates 1978–2006	261 710	9-year mandatory JMU programme rural immersion	Up to 28 years post-graduation for the earliest cohorts	JMU graduates are 4 times more likely to practice in a rural area after their obligation; OR: 4.2 (95% CI: 3.9–5.9)	Rural origin, primary care specialisation, post-graduate training in community hospitals, mandatory duration of rural practice, contract system.	2b

McGrail et al.[14]	2018	Australia	Retrospective longitudinal observational study	Medical graduates in Victoria 2008–2016	2 451	Prolonged (12- to 24-month) rural immersion	1- to 9-years post-graduate	Of the graduates who underwent rural training, 25% (90 of 357) returned to practice in the same rural region. Graduates with ≥12 months of rural training were 3.24 times more likely to settle in a rural area post-graduation than those with no rural training (based on multinomial regression analysis) Total number of graduates trained in rural areas who settled in rural areas (same or other region): 184 graduates = 26.2% of the 702 trained in rural areas. Graduates who completed ≥12 months of training in rural areas are 3.24 times more likely to settle in rural areas after graduation than those who did not receive rural training.	Duration of rural exposure (18–24 months), secondary schooling and training in the same rural region, rural origin.	2b
Tate & Aoki[15]	2012	Canada	Questionnairebased crosssectional study	University of Manitoba medical graduates 1965–2000	1 269	Rural immersion during medical school and residency	No longitudinal follow-up; retrospective study of career paths 1965–2000	Graduates with rural experience during medical school (UG) were 1.34 times more likely to have practiced in a rural area during their career; OR: 1.34 (95% CI: 1.09–1.75).	Male gender, rural origin, rural high school, rural experience during medical school and residency, duration and remoteness of rural exposure (greater distance from urban centres associated with higher rural practice rates).	2b
Wendling et al.[16]	2016	USA	Retrospective observational cohort study	Michigan State University Rural Physician Program (RPP) graduates 1978–2006	2778	2-year comprehensive rural clinical training	Up to 33 years for the earliest 1978 graduates	RPP graduates are approximately 3 times more likely to practice rurally than other MSU-CHM graduates; OR: 3.09 (95% CI: 2.12–4.50).	Rural origin, selection of students interested in rural medicine, 2-year rural clinical exposure, regular mentoring, enhanced family medicine training.	2b

Cortie et al.[17]	2023	Australia	Longitudinal cohort study	University of Wollongong medical graduates 2010–2021	22 807	Rural-focused training and rural clinical placements	Up to 12 years post-graduation	Wollongong graduates were 1.51 times more likely to practice rurally than others RR: 1.51 (95% CI: 1.34–1.71)	Rural origin, completion of a rural placement, older age, selection strategy favouring rural students.	2b
Playford et al.[18]	2015	Australia	Retrospective cohort study	Western Australia University graduates 1980–2011	324	Rural Clinical School of Western Australia (RCSWA), prolonged rural immersion in Year 5	3 to 34 years follow-up	78.7% of RCSWA graduates practice in remote rural areas. OR: 4.42 (95% CI: 2.26–8.67). Long-term rural training increases by 4.42 the probability of settling in a remote rural area.	Participation in a longitudinal rural clerkship (RCSWA), exposure to very remote sites (RA 3–5), female gender, independent effect of place of origin.	2b
Pagaiya et al.[19]	2015	Thailand	Retrospective cohort study	Graduates of the CPIRD programme 2000–2007	7 157	CPIRD Programme, a medical training with 3-year obligation of rural hospital practice	Followed for 11 years	29% of CPIRD physicians settled in rural areas. Standard track physicians have a 1.3 times higher risk of leaving rural areas than CPIRD physicians; HR: 0.795.	Recruitment of students from rural origins, mandatory return to the province of origin, 3year mandatory public service.	2b
Young et al.[20]	2011	Australia	Longitudinal study	John Flynn Placement Program JFPP medical students 2005–2009	1863	4-year JFPP repeated rural immersion (2 weeks/year)	Up to 8 years (4 years during training plus post-graduate follow-up until specialisation)	After a single JFPP placement, 9% of students intend to become rural GPs. After four JFPP placements, this proportion doubles to 19%. 65% of students who completed four JFPP placements express a desire for rural practice.	Repeated exposure to rural mentoring over 4 years, social and medical immersion, sense of community connection, progressive increase in rural GP intention.	2b

MacDowell et al.[21]	2013	USA	Retrospective comparative cohort study	Rural Medical Education Programme graduates 1997–2007	2823	16-week RMED programme targeted rural training in Year 4	Up to 15 years post-graduation; mean 5.3 years in practice (0.5–11.3 years)	6.3% of RMED graduates practice in rural areas (RUCA 4+), compared with 6.9% of non-RMED graduates. OR: 17.20 (95% CI: 12.18–24.35).	Rural background, participation in a rural-targeted medical programme, curriculum content (rural and indigenous health), prolonged rural placements, rural post-graduate training, career choice in general practice.	2b
Woolley et al.[22]	2021	Australia	Longitudinal observational cohort study	James Cook University graduates 2005–2014	900	20- to 35-week mandatory JCU rural placements in Year 6 plus rural GP/generalist postgraduate training	14 years followup	Graduates who completed an internship in a rural hospital are 54.6 times more likely to settle in a very remote area post-training; OR: 54.6 (95% CI: 4.4–678.1).	Rural origin, prolonged rural placements, JCU general practice training, MRBS scholarship, rural internship, general practice or generalist specialisation.	2b
Playford et Puddey[23]	2016	Australia	Retrospective cohort study	Western Australia graduates 2004–2010	868	10-month RCSWA rural immersion in a clinical year	3 to 10 years follow-up	20.7% of graduates who participated in the RCSWA programme work in a rural area; OR: 3.82 (95% CI: 2.46–5.93), P < .001. RCSWA participants are approximately 3.8 times more likely to work rurally than those who never expressed interest in a rural placement.	Participation in a rural immersion programme (RCSWA), age at graduation, rural origin, MRBS scholarship.	2b
Isaac et al.[24]	2014	Australia	Prospective longitudinal study	University of New South Wales medical students 2013	150	1-year or longer Australian Rural Clinical School (RCS) rural immersion	1 year of followup	Among those with an initial rural intention, only 6.3% changed to a metropolitan intention after one year. This indicates a net positive change in favour of rural practice.	Rural origin, duration of rural placement (>2 years), preference for a rural internship.	2b

Walker et al.[25]	2012	Australia	Cross-sectional observational study	Medical students participating Rural Clinical Schools in 6 universities 2006	125	RCS Programme, rural immersion during medical training	No longitudinal follow-up; postplacement data collected in 2006	42% (n=48) of students expressed a preference for rural practice.	Rural origin + RCS placement, chosen speciality (General Practice), change in attitude after rural experience, positive beliefs about rural life.	2b
Petrany et al.[26]	2017	USA	Retrospective comparative cohort study	Marshall University graduates in Family Medicine 1997–2014	138	Continuous free clinic experience during residency (1 half-day/week)	Up to 17 years	63% (27 of 43) of residents who participated in the free clinic settled in a rural area. 56% (24 of 43) settled in underserved areas.	Voluntary participation in a free-clinic placement.	2b
Pacheco et al.[27]	2005	USA	Cross-sectional study	University of New Mexico Family Medicine residency graduates 1974–2004	152	1+2 year family medicine training with rural placements (1 year Albuquerque, 2 years rural)	30 years followup	65.1% of rural residency graduates settle in rural areas compared to 25.8% for those in the urban programme.	Rural training (1+2 years), minority ethnic origin, graduation from a local medical school, locum tenens programme, community engagement.	2b
Sen Gupta et al.[28]	2014	Australia	Longitudinal cohort study	James Cook University (JCU) medical graduates 2005–2011	536	JCU programme, regional medical training with prolonged rural placements	7 years follow-up	An urban-background student who completes a rural internship is 6.1 times more likely to settle rurally than if they had completed a metropolitan internship; OR: 6.1 (95% CI: 2.9–12.6).	Rural origin, rural internship location, prolonged exposure to rural teaching, general practice specialisation.	2b
Jones et al.[29]	2000	USA	Cross-sectional comparative observational study	Morehouse School of Medicine graduates 1988–1997	263	4-week mandatory Rural Primary Care Clerkship (RPCC) in Year 3 or 4	No longitudinal follow-up; endof-studies questionnaire)	After the introduction of the mandatory rural placement, the percentage of students considering a rural career nearly doubled (from 11.8% to 20.0%). P-value: .094 (result not statistically significant).	Mandatory rural clinical exposure, community-based supervision.	3b

Straume & Shaw[30]	2010	Norway	Retrospective longitudinal cohort study	Medical interns who completed their placement in Finnmark 1999–2006	233	18-month Finnmark internship (mandatory training including 6 months in rural primary care with tutoring)	Up to several months following internship	University of Tromsø graduates are 3.6 times more likely to settle in the North after their placement than those from southern universities; OR: 3.6 (95% CI: 1.1–11.8).	Childhood location (specifically growing up in the North), university of graduation (Tromsø), rural internship experience (Finnmark), professional and social support during placement.	3b
Sen Gupta et al.[31]	2013	Australia	Retrospective comparative study	James Cook University (JCU) medical graduates 2005–2010	292	20-week or longer JCU rural clinical placements and regional immersion throughout the curriculum	Intention data collected at the end of the course (no postgraduation longitudinal follow-up)	Intention to settle rurally (JCU vs other schools); OR: 16.5 (95% CI: 11.3–24.0).	Rural origin of students, targeted selection, rural medicine-focused curriculum, high exposure to rural placements, positive placement experiences.	2b
Rourke et al.[32]	2018	Canada	Retrospective longitudinal observational study	Memorial University of Newfoundland medical graduates 2011–2020	698	Prolonged rural medical training programme and rural family medicine residency	Follow-up of entire pathway (pre-MD, MD, residency) to post-residency post	26.9% of family physicians trained at Memorial (MUN) practice in rural areas 2 years post-residency (2004–2013 cohort), compared to a national average of 13.3%.	Rural origin, inclusive admissions, rural curriculum, rural post-graduate training, rural professional development, local mentorship.	2b
Straume & Shaw[33]	2010	Norway	Descriptive longitudinal study	Medical interns who completed their placement in Finnmark 1999–2006	267	Finnmark primary care internship programme with tutoring and postgraduate training	5 years follow-up	Of the 267 medical interns, nearly twice as many as expected accepted full-time positions in Northern Norway following their training.	Rural post-graduate training, professional opportunities, peer support, family roots, practical learning in real-world contexts.	2b

Lewis et al.[34]	2016	Australia	Longitudinal study with questionnaire	NSW Rural Resident Medical Officer Cadetship Program graduates 1989–2010	142	Scholarship programme linked to a rural service commitment: financial support for studies + 2-year obligation in a rural hospital	Up to 25 years	53% of former cadets (n=74/140) were practising rurally (RA2–RA5) in 2014.	Post-graduate rural exposure, mentored training, rural origin, attractiveness of the return-ofservice scholarship programme.	2b
Playford et al.[35]	2015	Australia	Longitudinal cohort study	Rural Clinical School of Western Australia (RCSWA) graduates 2002–2013	367	1-year prolonged rural immersion across 14 rural towns	Up to 10 years post-graduation	39% of graduates had practised rurally at least temporarily by 2013; 17% were working fulltime in rural areas in 2013; 44% (183/417) were still in rural areas after 10 years.	Prolonged rural immersion, professional mobility encouraged, link with rural practice.	2b
Playford et al.[36]	2017	Australia	Retrospective cohort study with multivariate analysis	University of Western Australia medical graduates 2006–2010	569	1-year immersion in Rural Clinical School (RCS) in the final year of training	2 to 5 years postgraduation	The RCS placement alone slightly increases entering rural practice (OR: 1.93; 95% CI: 1.05–3.54), but the effect is significant primarily among students with an initial rural intention (OR: 3.57; 95% CI: 1.25–10.2).	Rural origin, initial intention of practising rurally, rural immersion experience during studies.	2b
Phillips et al.[37]	2017	USA	Retrospective observational study	Michigan State University College of Human Medicine graduates 1972–2006	3 107	Upper Peninsula (UP) campus: 2year prolonged rural placement in Years 3 and 4	Between 5 and 39 years	Entering rural practice (UP campus vs other campuses); OR: 4.42 (95% CI: 3.02–6.48). Entering HPSA zone practice (UP campus vs other campuses); OR: 1.28 (95% CI: 1.01–1.61).	Training in community campuses, prolonged exposure to local communities, roots in rural areas.	2b

Phillips et al.[38]	2013	USA	Retrospective longitudinal observational study	Medical residents trained in Federally Qualified Health Centers (FQHC), Rural Health Clinics (RHC) or Critical Access Hospitals (CAH), 2001–2005	1 378	Training in underserved medical settings with clinical primary care imme rsion	4 to 8 years from the start of training	Rate of entering rural practice in 2009: CAH: 52.6% (72/137); RHC: 38.1% (205/538); FQHC: 31.2% (219/703).	Residency training in underserved care centres (FQHC, RHC, CAH).	2b
Woolley et al.[39]	2017	Australia	Cross-sectional study based on longitudinal graduate database	James Cook University medical graduates 2005–2011	529	Rural medical training programme with prolonged placements and rural family medicine residency	4 to 10 years post-graduation	Doctors with rural general practice training were 17 times more likely to be practising rurally for at least 1 year in a very remote area (RA 4–5); OR: 17.0 (95% CI: 6.9–42.1; P < .001). Those who completed an internship in an outer-regional or remote area (RA 3–5) were 3.5 times more likely to settle in a very remote area; OR: 3.5 (95% CI: 1.4–8.3; P = .006).	Rural medical training, high selection interview score, Darwin clinical school placement, female gender, rural or remote internship, minority group membership.	2b
Dick et al.[40]	2011	USA	Retrospective cohort study	Internal medicine residency graduates from a large academic centre 1996–2006	451	1 to 2 months rural training experience during Year 2 or 3 of residency	11 years (1996–2006) retrospective period; no post-residency follow-up	Residents who completed a rural placement were 2.1 times more likely to choose a career in primary medicine; OR: 2.1 (95% CI: 1.3–3.4).	General practice pathway, participation in a rural placement, male gender (negatively associated), more recent graduation year (negatively associated).	2b

Forster et al.[41]	2013	Australia	Retrospective online questionnaire study	University of New South Wales Rural Clinical School (UNSW RCS) graduates 2003–2010	214	Rural immersion of 1 to 3 years within the UNSW RCS programme	Graduates surveyed between 2003 and 2010 (no longitudinal follow-up, selfreported posttraining data)	26% of graduates currently work in rural areas. The rate of practising rurally varies by rural training duration: 1 year: 15% (6/40); 2 years: 23% (23/100); 3 years: 37% (25/67).	Duration of rural placement (3 years > 1 year), entry via rural stream, voluntary choice of rural placement.	2b
McGrail & O'Sullivan[42]	2021	Australia	Cross-sectional observational study from a national survey (MABEL)	Doctors who participated in rural medical training 2017	4097	Rural medical training of varying durations (<12 weeks, 3–12 months, >1 year)	Cross-sectional analysis in 2017 only	Doctors with 3–12 months of rural training were 1.42 times more likely to work in the same rural region as their training compared to those with <12 weeks; RRR: 1.42 (95% CI: 1.08–1.88). This increased to a RRR of 5.22 (95% CI: 3.95–6.89) for those with >1 year of training in the same region.	Rural origin, long-term training (>1 year) in the same rural region, combination of origin + training in the same region.	3b
Fielding et al.[43]	2022	Australia	Cross-sectional study	Early-career General Practitioners (6 months to 2 years postfellowship)	319	General practice training with rural placements (≥13 weeks in MMM2–7 zones)	No longitudinal follow-up	Doctors who completed at least 13 weeks of rural training (MMM2–7) were 16 times more likely to be entering rural practice post-training; OR: 16.0 (95% CI: 6.79–37.9; P < .001).	Rural training during internship, rural schooling preuniversity, having worked in the practice during training, having an unemployed spouse, training via a regional rural body.	2b
Putri et al.[44]	2021	Indonesia	Cross-sectional study	Early-career doctors (1 to 5 years postinternship)	3 176	Clinical placements and internships in rural and remote areas	No longitudinal follow-up	Doctors who completed a clerkship in an isolated rural area were 2.17 times more likely to work in a remote area post-training; OR: 2.17 (95% CI: 1.07–4.40; P < .05). Doctors who completed their internship in an isolated rural area were 1.96 times more likely to be practising rurally post-training; OR: 1.96 (95% CI: 1.29–2.96; P < .05).	Rural origin, placement or internship in a remote area, participation in a rural incentive programme, previous rural work experience, male gender, single status.	2b

Taylor & Goletz[45]	2016	USA	Retrospective cohort study	Year 3 medical students from Indiana University School of Medicine 2009–2014	587	4-week family medicine clerkship funded by Area Health Education Centers (AHEC)	No longitudinal follow-up	Students who completed their placement rurally were more likely to report an intention of entering rural practice (21.6% vs 12.3%). Students in AHEC-supported placements were not significantly more likely to report a rural career intention (18.8% vs 13.1%).	Rural placement experience, partnerships with AHEC	2b
Walker et al.[46]	2021	Australia	Retrospective cohort study	Flinders University medical graduates 1999–2012	1 211	Parallel Rural Community Curriculum (PRCC) – prolonged rural immersion in general practice	From end of training until workplace analysis in April 2017	In 2017, based on selfreporting, PRCC graduates were significantly more likely to be practising rurally compared to urban graduates: 2.2 times more likely in MM 2–7 (OR: 2.2; $P < .001$), 3.1 times more likely in MM 3–7 (OR: 3.1; $P < .001$), and 3.4 times more likely in remote MM 5–7 areas (OR: 3.4; $P < .0001$).	Participation in a rural training programme (PRCC), particularly in general practice.	2b
Moffatt[47]	2017	Australia	Cross-sectional observational study with selfadministered questionnaire	University of Queensland medical students 2007–2011	1 020	Rural Medicine Rotation (RMR) – compulsory 8week clinical placement	No longitudinal follow-up	Students who felt a strong sense of clinical responsibility rurally were 3.55 times more likely to report that their placement positively influenced their rural intention; OR: 3.55 (95% CI: 2.05–6.14). Students who experienced managing common health issues were 2.16 times more likely to report positive intent; OR: 2.16 (95% CI: 1.43–3.28).	Understanding of rural clinical responsibilities, interprofessional learning, clinical case diversity, consultation skill acquisition.	4

Crampton et al.[48]	2013	International	Systematic review	Studies on clinical placements in underserved areas 1990–2021	54 studies included	Medical placements in rural and medically underserved areas	6-year follow-up	18 studies showed a positive association between rural clinical placements and practising rurally.	Prolonged exposure in underserved areas improves clinical competence, confidence, community attachment, rural background.	2b
Dahal et al.[49]	2023	USA	Retrospective cohort study	University of Washington medical graduates 2009–2014	994	4-week placement in rural primary care: Rural Underserved Opportunities Program (RUOP)	Approximately 9 to 14 years post-RUOP	RUOP participants were 1.83 times more likely to be practising rurally; OR: 1.83 (95% CI: 1.05–3.13; P = .03). Adjusted OR: 1.77 (95% CI: 1.02–3.05; P = .04).	Participation in a rural placement (RUOP), initial interest in the rural region (WWAMI)*, preference for family medicine and outpatient practice, rural origin, lack of interest in tertiary care.	2b
Jones et al.[50]	2014	Australia	Prospective longitudinal study	Australian medical school graduates 2005– 2008	3 268	Rural clinical placements (Years 3–4) vs urban placements	Medical school entry to graduation (2005–2011)	Students with late rural placements were 1.32 times more likely to intend entering rural practice; OR: 1.32 (95% CI: 1.00–1.74; P = .051). Remote placements had a stronger effect; OR: 1.77 (95% CI: 1.25–2.49; P = .001). Conversely, late urban placements significantly reduced rural intention; OR: 0.47 (95% CI: 0.32–0.69; P < .0001).	Placement duration and timing (especially late-stage), school type (rural vs urban), rural origin, initial intent, rural clubs or scholarships.	2b

Ogden et al.[51]	2020	Global (mainly Australia, USA, and Canada)	Systematic review and metaanalysis	GPs who received rural medical training	Included 27 studies	Rural experiences during medical school and postgraduate training	From several months to 31 years posttraining	Undergraduates with rural training were 1.75 times more likely to be practising rurally; OR: 1.75 (95% CI: 1.48–2.08). Post-graduates (interns/residents) with rural training were 4.57 times more likely to work rurally; OR: 4.57 (95% CI: 2.80–7.46).	Rural origin, undergraduate rural placement, post-graduate rural training, cumulative effect of rural experiences, placement duration, final-year rural training.	1a
Seal et al.[52]	2022	Australia	Longitudinal cohort study	2011 medical graduates from 10 Australian universities with rural/regional clinical schools	1 321	Prolonged Rural Clinical School (RCS) experience of at least 1 year	Cohort study 2011 domestic medical graduates from ten Australian medical schools with rural clinical or regional medical schools.	Urban-origin graduates with a prolonged RCS placement were 2.7 times more likely to be practising rurally 8 years after graduation; RR: 2.7 (95% CI: 1.8–4.2) compared to urban graduates without RCS training.	Rural origin, prolonged RCS placement, combination of both, retention strengthened by prolonged rural experience.	2b
Johnson et al.[53]	2018	International	Systematic review	Rural medical education programmes 2005–2017	Included 62 studies	Rural clinical placement programmes with or without specific curricula	Up to 25 years	43 studies indicate an increase in graduates practising rurally after rural training. Longer placements (6 to 12 months) are more effective than short ones (<12 weeks). Rural-origin students are more likely to enter rural practice posttraining.	Rural origin, initial rural intentions, duration of rural placement exposure, interest in general practice or primary care, voluntary participation in structured and financed programmes, cumulative effect of prolonged experiences.	1a
McGrail et al.[54]	2023	Australia	Cohort study	University of Queensland medical graduates 2012–2021	2 806	Rural placements of 6 to 12 weeks, with or without prolonged regional training	Up to 10 years	Participants in prolonged rural placements (12 weeks) were 2.2 times more likely to be practising rurally; OR: 2.2 (95% CI: 1.1–4.6). Effect is strongest for those combining rural immersion with prolonged regional training; OR: 8.6 (95% CI: 4.5–16.3).	Prolonged placement duration (12 weeks), rural or remote setting, rural origin, female gender, combination with long regional training (1 to 2 years), self-selection.	2b

Williams et al.[55]	2023	Australia	Retrospective cohort study	University of Adelaide medical graduates 2004–2019	2 078	Prolonged rural placement of 36 weeks (full clerkship year in Year 5)	Up to 16 years post-graduation	Rate of entering rural practice (MMM3–7): ARCS** (prolonged rural training): 14.7%; Urban training: 5.3%. Adjusted OR: 3.06 (95% CI: 2.06–4.53); ARCS students are 3 times more likely to enter rural practice.	Prolonged rural training (36 weeks), rural origin, post-graduate seniority (PGY4+), self-selection of motivated students, local impact of the training site.	2b
Connell et al.[56]	2022	New Zealand	Prospective cohort study	University of Auckland medical graduates 2009–2018	1313	Northland Regional-Rural Admission Scheme (Pūkawakawa): 1 full year with 20 weeks of regional hospital placements and 7 weeks of integrated rural GP care	3 to 12 yeas postgraduation	Pūkawakawa graduates were 3.4 times more likely to return to work in Northland; RR: 3.4 (95% CI: 1.9–6.2). They were also more likely to be practising rurally in other regions, though the effect was smaller; RR: 1.4 (95% CI: 0.9–2.2).	Participation in a regional-rural immersion programme (Pūkawakawa), rural or regional origin, ethnicity, relational and identity-based effects associated with the placement site.	2b
Abid et al.[57]	2020	New Zealand	Cross-sectional observational study	University of Auckland and Otago medical graduates 2011–2017	241	Long (>33 weeks) and short (5 weeks) rural immersion programmes	Baseline and end-of-course questionnaires (approximately 6 years)	Students with a long rural placement (>33 weeks) were 6.4 times more likely to report a rural intention; RR: 6.4 ($P < .001$). They were also 4.4 times more likely to report an intent to practice regionally; RR: 4.4 ($P < .001$).	Long rural immersion (>33 weeks), initial rural intention, rural or regional origin, rural club membership, preference for general practice.	2b

Williamson et al.[58]	2012	New Zealand	Retrospective cohort study with longitudinal follow-up	University of Otago medical graduates 2000–2001	147	7-week rural placement in Year 5 (Dunedin)	8 to 9 years postplacement	6% of exposed students (2/36) and 9% of non-exposed (10/111) were practising rurally at follow-up. No significant difference was found between groups ($P = .155$).	Prolonged immersion, positive perception of teachers regarding rural medicine.	2b
Eley et al.[59]	2009	Australia	Prospective longitudinal cohort study	Graduates from several Australian Rural Clinical Schools (RCS) 2009–2014	124	Prolonged RCS placements	1 to 5 years postgraduation	29% of respondents were practising rurally (RRMA 3–7).	Duration of rural exposure (2 years vs 1 year), rural origin, rural lifestyle, mentorship, autonomy in placement organisation, suitable specialties (GP, emergency).	2b
Williams et al.[60]	2023	Australia	Retrospective cohort study	Students having completed 1 full year in Adelaide Rural Clinical School 2013–2018	234	Integrated 36-week rural placement in Year 5	1 to 6 years postgraduation	11.5% (95% CI: 8.0–16.3) were practising rurally (MMM 3–7 classification); 16.7% (95% CI: 12.4–22.0) using ASGS 2–5 classification.	Rural origin, longest place of residence being rural, stated preference for rural practice, self-efficacy scores for rural practice.	2b

O'Sullivan et al.[61]	2018	Australia	Longitudinal observational cohort study	Monash University medical graduates 2008–2016	2 412	Rural immersion of varying durations and contexts in Years 3–5	Up to 9 years	1-year immersion significantly increased the odds of practising rurally; OR: 1.79 (95% CI: 1.15–2.79). 1 to 2 years: OR: 2.26 (95% CI: 1.54–3.32). ≥ 2 years: OR: 4.43 (95% CI: 3.03–6.47).	Duration of rural immersion (dose-response effect), combination of regional hospital and rural GP placements, rural origin, service obligations, international student status.	2b
Seal et al.[62]	2024	Australia	Longitudinal observational cohort study	2011 medical graduates from 9 Australian universities (FRAME members)	1 220	Rural Clinical School (RCS) experience	10 years	At PGY10, 19% of specialists were practising rurally (MM2–7). By specialty: GPs: 28.2%; Non-GP specialists: 12.3% (medical: 10%; surgical: 15%).	Rural origin, prolonged undergraduate rural placements, specialty choice (GP vs non-GP), rural specialist training opportunities.	2b
Playford et al.[63]	2014	Australia	Retrospective cohort study	University of Western Australia medical graduates 2002–2009	1 017	Rural Clinical School of WA (RCSWA) 1-year placement in Year 5	4 to 11 years post-placement	Rate of entering rural practice: RCSWA: 16.3% (42/258); Controls: 4.7% (36/759). Adjusted OR: RCSWA + rural background: 7.5; RCSWA + urban background: 5.1.	RCSWA participation, rural origin, female gender, age ≥40, strong effect for urban students following RCS placement.	2b

Eley & Baker[64]	2009	Australia	Descriptive observational study	Year 3 University of Queensland medical students	463	Compulsory 8week Rural Medicine Rotation (RMR)	No longitudinal follow-up	76% reported the rotation encouraged interest in rural medicine; effect more pronounced in urban-origin students.	Prolonged rural exposure, clinical confidence, understanding rural practice, rural mentorship, academic/logistical support, active learning.	3b
Woloschuk & Tarrant[65]	2002	Canada	Prospective prepost observational study with questionnaire	Year 3 University of Calgary medical students	254	Compulsory 4week rural family medicine rotation	Immediate pre- and post-rotation	Both urban and rural students reported a significant increase in intent for rural locums postplacement: $P < 0.008$. No significant increase in intent for long-term rural practice; $P > .05$.	Experience increases rural locum intent; rural origin; no gender effect; stronger effect in family medicine-oriented students.	2b
Tavernier et al.[66]	2003	USA	Cross-sectional questionnaire study	Year 3 family medicine residents	608	Exposure to Medically Underserved Areas (MUA)	No longitudinal follow-up	36% (285/608) chose to enter practice in an MUA. Prior MUA experience increased the likelihood by 1.5 times; OR: 1.5 (95% CI: 1.1–2.3).	Rural origin, exposure to MUAs during college and medical school, ethnic minority status, medical service obligations.	2b

*WWAMI is a regional medical education partnership between the University of Washington School of Medicine and the states of Washington, Wyoming, Alaska, Montana, and Idaho. It is designed to provide medical education and clinical training specifically tailored to the needs of rural and underserved communities across these five states.

**ARCS is the Adelaide Rural Clinical School, synonymous with a prolonged rural placement.
