# Supplementary material for: The Impact of Rural Clinical Placements on Medical Students’ Career Choices: A Systematic Review
Source: Int J Health Policy Manag. 2026 May 5;15:9328. doi: 10.34172/ijhpm.9328 (PMC13498956; doi:10.34172/ijhpm.9328)
Supplement: Supplementary file 3 [file ijhpm-15-9328-s003.pdf]

Article title: The Impact of Rural Clinical Placements on Medical Students' Career Choices: A Systematic Review

Journal name: International Journal of Health Policy and Management (IJHPM)

Authors' information: Marc-Antoine Schirm[1], Jan Chrusciel[2], Maxime Thorigny[2], Aline Hurtaud[3], Nathalie Bednarek[4], Stephane Sanchez[1,5]*

[1]Public Health Department, Champagne Sud Hospital, Troyes Hospital, Troyes, France.

[2]Department of Neonatology, Reims University Hospital Alix de Champagne, Reims, France.

[3]Department of General Practice, Faculty of Medicine, University of Reims Champagne-Ardenne, Reims, France.

[4]Laboratory REGARDS, Research Unit 6192, University of Reims Champagne-Ardenne, Reims, France.

[5]Research Unit 3797 VieFra, Faculty of Medicine, University of Reims Champagne-Ardenne, Reims, France.

***Correspondence to:** Stephane Sanchez; Email: stephane.sanchez@hcs-sante.fr

Citation: Schirm MA, Chrusciel J, Thorigny M, Hurtau A, Bednarek N, Sanchez S. The impact of rural clinical placements on medical students' career choices: a systematic review. Int J Health Policy Manag. 2026;15:9328. doi:10.34172/ijhpm.9328

Supplementary file 3. Funnel Plot for Evaluating Publication Bias

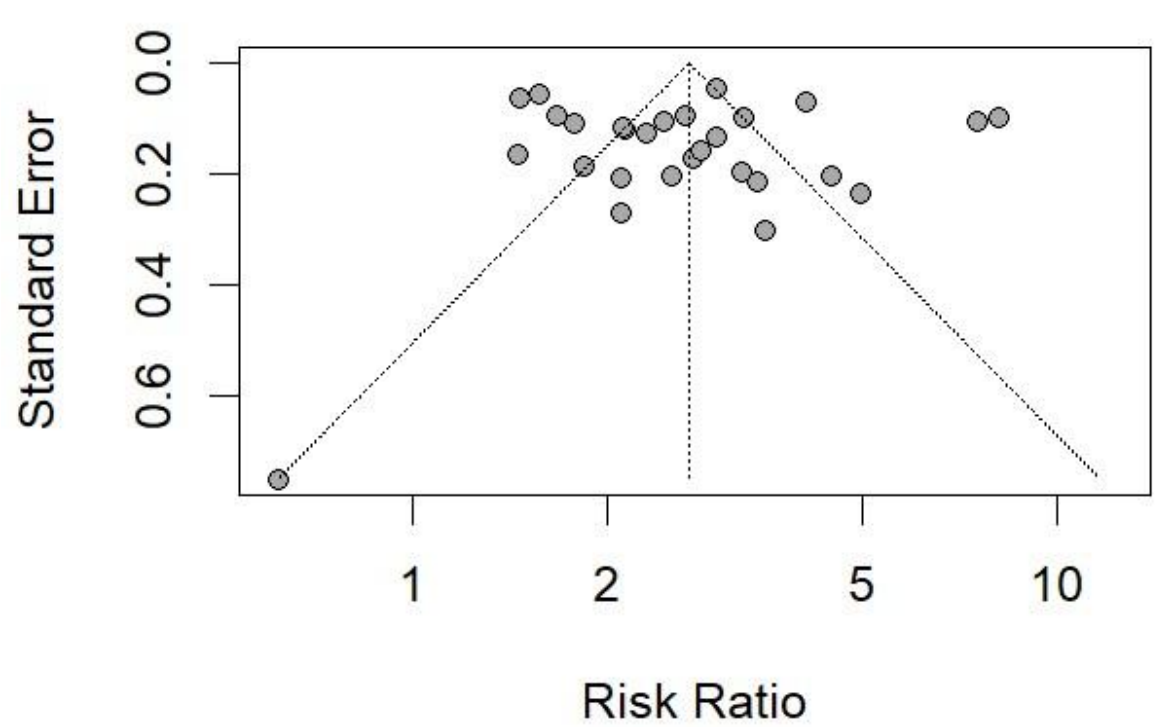

The funnel plot shows moderate asymmetry, with small studies reporting larger effect sizes more often represented than small studies reporting weaker or null effects. This pattern may suggest the presence of a publication bias or small-study effect. However, given the high heterogeneity observed across studies (I^2 = 94.9%), asymmetry must be interpreted with caution, as heterogeneity alone can generate funnel plot distortion. While a publication bias cannot be entirely excluded, it does not invalidate the overall signal but may indicate that the pooled estimate is slightly overestimated.
